# Visualization of postoperative anterior cruciate ligament reconstruction bone tunnels: Reliability of standard radiographs, CT scans, and 3D virtual reality images

**DOI:** 10.3109/17453674.2011.623566

**Published:** 2011-11-25

**Authors:** Duncan E Meuffels, Jan-Willem Potters, Anton HJ Koning, Charles H Brown Jr, Jan AN Verhaar, Max Reijman

**Affiliations:** ^1^Department of Orthopaedic Surgery; ^2^Department of Bioinformatics, Erasmus MC, University Medical Center Rotterdam, the Netherlands; ^3^Abu Dhabi Knee and Sports Medicine Center, Abu Dhabi, United Arab Emirates

## Abstract

**Background and purpose:**

Non-anatomic bone tunnel placement is the most common cause of a failed ACL reconstruction. Accurate and reproducible methods to visualize and document bone tunnel placement are therefore important. We evaluated the reliability of standard radiographs, CT scans, and a 3-dimensional (3D) virtual reality (VR) approach in visualizing and measuring ACL reconstruction bone tunnel placement.

**Methods:**

50 consecutive patients who underwent single-bundle ACL reconstructions were evaluated postoperatively by standard radiographs, CT scans, and 3D VR images. Tibial and femoral tunnel positions were measured by 2 observers using the traditional methods of Amis, Aglietti, Hoser, Stäubli, and the method of Benereau for the VR approach.

**Results:**

The tunnel was visualized in 50–82% of the standard radiographs and in 100% of the CT scans and 3D VR images. Using the intraclass correlation coefficient (ICC), the inter- and intraobserver agreement was between 0.39 and 0.83 for the standard femoral and tibial radiographs. CT scans showed an ICC range of 0.49–0.76 for the inter- and intraobserver agreement. The agreement in 3D VR was almost perfect, with an ICC of 0.83 for the femur and 0.95 for the tibia.

**Interpretation:**

CT scans and 3D VR images are more reliable in assessing postoperative bone tunnel placement following ACL reconstruction than standard radiographs.

Non-anatomic bone tunnel placement has been reported to be the most common cause of a failed ACL reconstruction (Khalfayan et al. 1996, Zantop et al. 2007). Although the anatomic attachment sites of the ACL have been well described, the optimal bone tunnel placement for ACL grafts remains controversial. Given the importance of bone tunnel placement for the success of the procedure, radiographic methods to postoperatively assess bone tunnel placement would be helpful in documenting postoperative outcomes.

Recent studies have validated the use of 3D CT scans and MRI for evaluation of ACL bone tunnel placement postoperatively (Abebe et al. 2009, Forsythe et al. 2010). The authors have questioned the reliability of conventional radiographs to evaluate ACL bone tunnel placement (Forsythe et al. 2010).

MRI is a good imaging modality for direct visualization of the ACL graft (McCauley et al. 2003, Moon et al. 2008). However, there have been no studies on the reliability of MRI scans to document bone tunnel placement following ACL reconstruction. Recently, a new 3D viewing and measurement method was developed for visualization of the ACL reconstruction. This method uses CT data and an immersive virtual reality system. We evaluated the reliability of standard radiographs, CT scans, and a 3D VR approach for evaluation of ACL bone tunnel placement.

## Patients and methods

We prospectively evaluated 50 consecutive patients (mean age 27 (18–41) (6.9 SD) years, 38 men) who underwent a primary ACL reconstruction from January 2007 until May 2008 (trial number ISRCTN 40231111). Patients gave their written consent and permission to participate in the study and institutional approval for the study was granted by the Medical Review Board of our institute.

ACL reconstruction was performed using an arthroscopic, single-incision, single-bundle, transtibial surgical technique using either bone-patella tendon-bone (BPTB) or a looped semi-tendinosus, gracilis autograft. The femoral and tibial bone tunnels were positioned within the native anatomic ACL footprint. ACL reconstructions performed using a BPTB graft were fixed on both sides using a resorbable interference screw (BIORCI; Smith and Nephew, Andover, MA). Hamstring ACL reconstructions were fixed using an extracortical button technique (Endobutton; Smith and Nephew) on the femoral side and a resorbable interference screw on the tibial side (BIORCI).

### Imaging

Standard radiographs were taken 6 weeks postoperatively when the patient was able to bear weight fully and to fully extend the knee. The AP radiograph was taken with the patient bearing full weight on the operative knee. The lateral radiograph was taken with the knee in extension, with an optimal overlay of the femoral condyles.

A 64-channel multi-slice technology CT scanner (Somatom; Siemens Medical Solutions, Forchheim, Germany) with helical acquisition in 1.0-mm sections (120 kV, 160 mAs, rotation time 1.0 s) was used to perform CT scans. The knee CT imaging was performed from the top of the suprapatellar collection to the superior tibial and fibula diaphysis, one day postoperatively.

### Measurements

**Figure 1.**
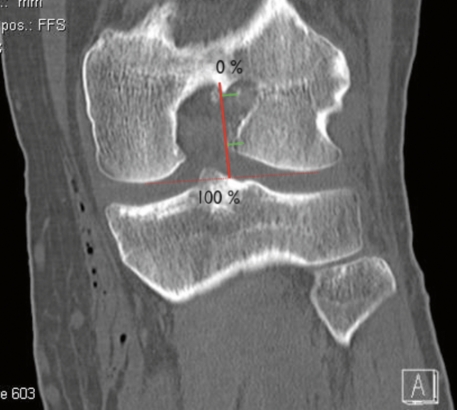
Measurement of the femoral tunnel on the coronal CT reconstruction, performed according to Hoser et al. (2005). The tunnel is measured in comparison to the line perpendicular to the most distal points of the femoral condyles. The measurement is compared to the line from the intracondylar roof to the distal femoral condyles.

**Figure 2.**
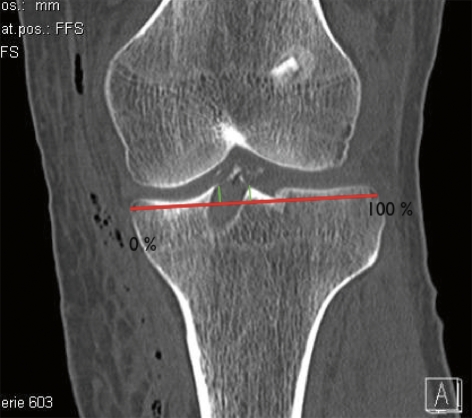
Medial-lateral measurement in the coronal CT reconstruction of the tibial tunnel. The tunnel measurement is compared to the line through the most medial and lateral part of the tibial plateau.

Measurements were performed digitally on all radiographs and CT slices. For all radiographic measurements, a tunnel was only rated as being visible if the tunnel and the necessary points to carry out the measurement were visible. In the AP image, we measured the femoral tunnel according to Hoser (Hoser et al. 2005) and the position of the tibial tunnel was measured as a percentage of the total tibial width from medial to lateral. These 2 measurements were also performed on coronal CT reconstructions (Figures 1 and 2).

**Figure 3.**
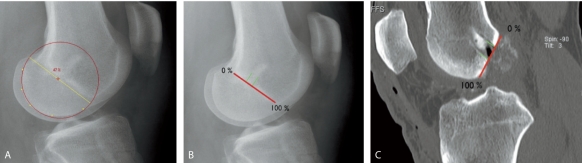
A. Lateral view of the lateral femoral condyle by the method of Amis (Amis et al. 1994). The yellow dots are the user-defined edges of the posterior femoral condyle. A circle is automatically fitted on to the dots and can be rotated, in such a way that the diameter is parallel to Blumensaat's line. B. Lateral view of the lateral femoral condyle by the method of Aglietti (Aglietti et al., 1995). These authors compare the tunnel measurement to a line parallel to the most posterior and anterior part of Blumensaat's line. C. Measurement according to Aglietti on the sagittal femoral CT reconstruction.

**Figure 4.**
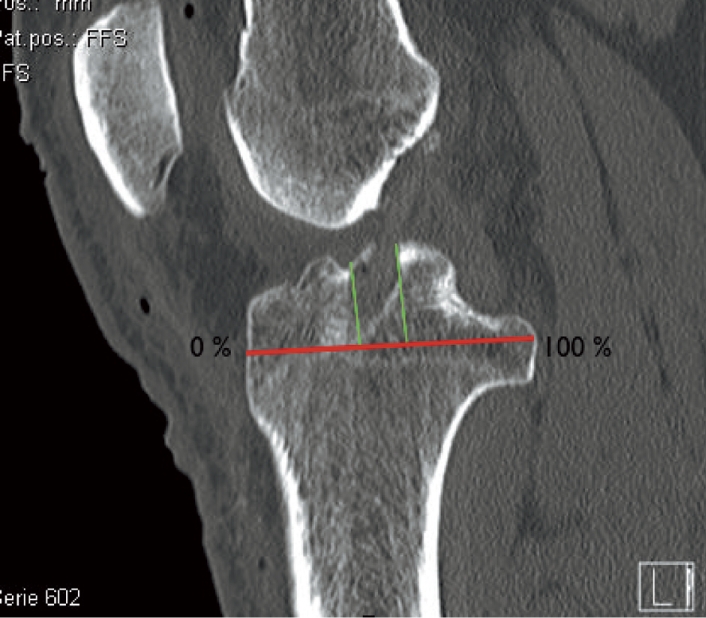
Measurement according to Stäubli (Stäubli and Rauschning, 1994) on the sagittal tibial CT reconstruction. The aperture of the tibial tunnel is compared to the line parallel to the joint line, through the most anterior and posterior points of the intracondylar tibial plateau.

On the lateral radiograph, the methods of Aglietti et al. (1995) and Amis et al. (1994) were used to measure the position of the femoral tunnel (Figure 3). On the sagittal CT images, the femoral tunnel was measured by the method of Aglietti since the Amis method is not feasible because Blumensaat's line and the femoral condyles are not in the same sagittal CT slice. The tibial tunnel position was measured as a percentage of the anterior to posterior tibia diameter in both the standard radiograph and in CT images according to method of Stäubli et al. (1994) (Figure 4).

Measurements were performed blind on images in such a way that each method was performed in random order for all patients before starting with another method. This protocol avoided the possibility that the observer could use the information from one measurement method in another method. 2 observers carried out all measurements independently. The experience of the observers in interpreting ACL reconstruction positioning images ranged from none with the 3D VR system to more than 12 years with the standard radiographs. After 6 weeks, all measurements were performed a second time by one observer to calculate intraobserver reproducibility. In the second sequence of CT measurements, the observer had to decide (again) the slide on which to perform the measurement.

### 3D virtual reality measurements

**Figure 5.**
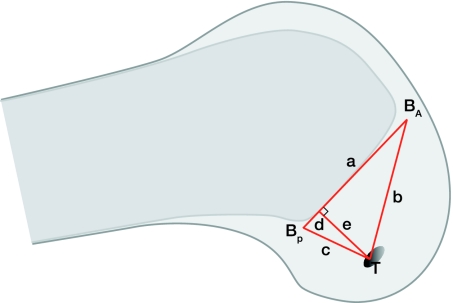
View of the lateral femoral condyle. The red triangle is used in the 3D visualization technique calculations. a = line equal to Blumensaat's line. BA = the most anterior point of Blumensaat's line. BP = the most posterior point. T = tunnel entrance. e = the line perpendicular to a, from the tunnel entrance. Lines b and c are the lines connecting T with Ba and Bp, respectively. This makes it possible to calculate the length of d, thus giving a percentage of the AP positioning of the tunnel position on the Blumensaat line comparable to Aglietti's method.

Measurements were performed using an I-Space immersive virtual reality system (I-Space; Barco NV, Kortrijk, Belgium) in 3D, which works similarly to the triangle method by Benareau (Figure 5) (Chouteau et al. 2007). The 3D VR approach uses a 4-sided immersive virtual environment where—with the aid of 8 projectors and polarizing glasses—the bony structures are projected as free-floating 3D objects in the room. The system uses the V-Scope direct volume-rendering software developed at our institute, and high resolution CT scans to visualize the bones (Koning 2009). Using a wireless joystick, it is possible to rotate the bones in three dimensions and point out distinctive points on the bony structure with a precision of 0.1 mm (Verwoerd-Dikkeboom et al. 2008).

### Statistics

Intraclass correlation coefficient (ICC) was calculated using the percentages of the different measurements. The calculation of the ICC is based on an analysis of variance (ANOVA) model. The first source of variance is the difference between the patients we measured. The second source of variability is the variance among the observers. The ICC calculations that were performed used the 2-way mixed model for absolute agreement. The ICC can be expressed on a scale from 0 to 1, where 0 expresses disagreement and 1 is perfect agreement. A score of 0.7 and higher is generally considered to be good in reliability studies (Nunnally 1994). We used chi-square test to determine the difference in radiographic visibility of the tunnels between the graft types.

## Results

### Tunnel visibility

CT allowed visualization of the femoral and tibial tunnels in both the AP and the lateral planes in all cases; visualization was less for the standard radiographs. It was more difficult to visualize the femoral tunnel (26/50) than the tibial tunnel (41/50) (p = 0.01). Femoral tunnel visibility on the lateral knee radiograph was lower in the ACL reconstruction with hamstring (4/16) than with BPTB (22/34) (p = 0.01).

### Interobserver reliability (Table)

In the AP radiographs, the ICC of the femoral method was 0.39 and that of the tibial was 0.43. On the lateral radiograph, the method of Amis gained the highest ICCs. The Amis method gave 0.62 as opposed to 0.53 for the method of Aglietti. The ICC for the tibial position on the lateral radiograph was 0.53.

**Table 1.** Intraclass correlation coefficients (ICCs)

	Interobserver	Intraobserver
	ICC (95% CI)	ICC (95% CI)
AP radiograph		
Femur (Hoser)	0.39 (–0.32 to 0.81)	0.47 (–0.12 to 0.83)
Tibia (med-lat)	0.43 (0.12–0.66)	0.43 (0.05–0.71)
Lateral radiograph		
Femur (Aglietti)	0.53 (0.03–0.82)	0.83 (0.50–0.95)
Femur (Amis)	0.62 (0.20–0.84)	0.72 (0.23–0.92)
Tibia (Stäubli)	0.53 (0.21–0.75)	0.82 (0.60–0.92)
CT coronal		
Femur (Hoser)	0.49 (0.25–0.68)	0.60 (0.39–0.75)
Tibia (med-lat)	0.76 (0.27–0.90)	0.90 (0.83–0.94)
CT sagittal		
Femur (Aglietti)	0.71 (0.47–0.84)	0.87 (0.79–0.93)
Tibia (Stäubli)	0.61 (–0.05 to 0.84)	0.63 (0.18–0.82)
3D Virtual Reality		
Femur (Benareau)	0.83 (0.70–0.90)	0.85 (0.75–0.92)
Tibia (Stäubli)	0.95 (0.92–0.97)	0.96 (0.93–0.98)

CT gave higher ICCs than the radiographs. The femur in the coronal plane gave the lowest ICC (0.49). The ICC of the tibia in the coronal plane (0.76) was considered good. The ICCs of the femur (0.71) and tibia (0.61) in the sagittal plane were in substantial agreement.

The 3D VR approach resulted in the highest interobserver ICCs of all methods: 0.83 for the femur and 0.95 for the tibia.

## Discussion

The 3D VR approach resulted in the highest ICCs and showed that measurement of the complex anatomy of the knee can be carried out reliably. The existing methods, using standard radiographs, showed a significantly lower visibility, especially regarding the use of the hamstring graft. At its best, the inter- and intraobserver agreement was substantial. CT showed optimal visibility, but only showed slightly better agreement—especially for the femur—because of its complex 3D shape.

Only 1 previous study has determined the reliability of measurements on the lateral femur (Klos et al. 2000). The authors reported an ICC of 0.68 with the method of Amis, but with fluoroscopically controlled placement before drilling of a femoral tunnel. The ICC found is in concordance with our findings, where the method of Amis produced the best ICC for radiographs (0.62). Furthermore, only one study has investigated the best modality (Hoser et al. 2005) and the authors concluded that there was no significant difference in the values of the tunnel position in the lateral femur measured on the radiograph and by CT. Based on our study, however, CT is more reliable than radiography in the lateral femur measurements.

Another method is the use of the clock, a popular reference method for intraoperative positioning of the femoral tunnel. It has been used in numerous studies, both clinical (Behrend et al. 2006) and anatomical (Amis et al. 1994, Giron et al. 2006). The method has certain disadvantages: there is no standardized clock shape or position, and it is very difficult to standardize the measurement since there are a number of variables that influence the measurement (Yoo et al. 2008). The variables to consider are (1) the position of the knee and its flexion angle and coronal positioning, (2) the viewpoint of the observer, and (3) the shape of the intercondylar space.

Conventional radiographs showed low ICC and also poor visibility, especially in the AP projection. We encountered a relatively low visibility rate compared to other studies, because we used a biologically resorbable fixation screw, which is not radio-opaque. Previous studies may have measured the metal interference screw position or the femoral aiming device position, which, however, need not correlate with the actual tunnel or graft position (Klos et al. 2000). In addition, the time between the surgery and radiography was short, so there were no sclerotic lines present, which could have helped to identify the position of the tunnel.

The CT scans gave more reliable measurements than the radiographs. However, one must bear in mind that CT scans are more expensive and that the patient is exposed to a higher dose of radiation. There has been growing interest in visualization of the ACL insertions and their relationship to bony landmarks using high-resolution volume rendering CT (Basdekis et al. 2009, Purnell et al. 2008). This technique uses the same CT images as in our study, but visualization and measurement is done on a computer screen. This measurement, however, limits the measurements to the 2 dimensions of the screen being viewed and probably makes it more susceptible to positioning inaccuracies, as shown in the moderate ICCs obtained for our CT measurements.

Based on the present study, the 3D VR approach is the most reliable system for performing measurements on the reconstructed ACL. The possibility to visualize the bone from any desired position allows many possibilities for measurement of other distances also, and for evaluation of anatomy. However, the use of virtual reality solutions to evaluate patients in clinical practice remains somewhat futuristic, and this method has not yet been introduced in clinical practice. The disadvantages of an immersive virtual reality system are, for example, that the system is expensive and labor-intensive. At present, a tabletop VR system offering the same functionality is being developed to overcome some of the disadvantages mentioned above.
